# Simultaneous mapping of metabolites and individual macromolecular components via ultra‐short acquisition delay ^1^H MRSI in the brain at 7T

**DOI:** 10.1002/mrm.26778

**Published:** 2017-06-22

**Authors:** Michal Považan, Bernhard Strasser, Gilbert Hangel, Eva Heckova, Stephan Gruber, Siegfried Trattnig, Wolfgang Bogner

**Affiliations:** ^1^ High Field MR Center, Department of Biomedical Imaging and Image‐guided Therapy Medical University Vienna Vienna Austria; ^2^ Christian Doppler Laboratory for Clinical Molecular MR Imaging Vienna Austria

**Keywords:** macromolecules, parameterization, human brain, MR spectroscopic imaging

## Abstract

**Purpose:**

Short‐echo‐time proton MR spectra at 7T feature nine to 10 distinct macromolecule (MM) resonances that overlap with the signals of metabolites. Typically, a metabolite‐nulled in vivo MM spectrum is included in the quantification`s prior knowledge to provide unbiased metabolite quantification. However, this MM model may fail if MMs are pathologically altered. In addition, information about the individual MM peaks is lost. In this study, we aimed to create an improved MM model by parameterization of the in vivo MM spectrum into individual components, and to use this new model to quantify free induction decay MR spectroscopic imaging (FID‐MRSI) data.

**Methods:**

The measured in vivo MM spectrum was parameterized using advanced method for accurate, robust, and efficient spectral fitting (AMARES) and Hankel‐Lanczos singular value decomposition algorithms from which six different MM models were derived. Soft constraints were applied to avoid over‐parameterization. All MM models were combined with simulated metabolite spectra to form complete basis sets. FID‐MRSI data from 14 healthy volunteers were quantified via LCModel, and the results were compared between all basis sets.

**Results:**

The MM model using nine individual AMARES‐parameterized MM components with additional soft constraints achieved the most reliable results. Nine MMs and seven metabolites were mapped simultaneously over the whole slice.

**Conclusion:**

The proposed MM model may facilitate studies that involve patients with pathologically altered MMs. Magn Reson Med 79:1231–1240, 2018. © 2017 The Authors Magnetic Resonance in Medicine published by Wiley Periodicals, Inc. on behalf of International Society for Magnetic Resonance in Medicine. This is an open access article under the terms of the Creative Commons Attribution License, which permits use, distribution and reproduction in any medium, provided the original work is properly cited.

## INTRODUCTION

Proton MR spectroscopic imaging (^1^H‐MRSI) is a powerful, noninvasive technique that provides valuable insights into brain metabolism. ^1^H‐MRSI benefits from the increased chemical shift dispersion and signal‐to‐noise ratio (SNR) at ultra‐high magnetic field strengths ( ≥ 7T). To take full advantage of the ultra‐high field, ultra‐short acquisition delay (TE*) free induction decay (FID)‐MRSI sequences recently have been developed 1, 2, 3, 4. With FID‐MRSI, relaxation‐related SNR losses are minimized and J‐coupling evolution is eliminated, thus allowing the quantification of more metabolites compared to what is available at lower fields ( ≤ 3T) 5. However, MR spectra measured with such sequences contain prominent high‐molecular‐weight macromolecules (MMs) signals superimposed on the signal of low‐molecular‐weight metabolites. These MM contributions are particularly strong due to their short T_1_ and T_2_ relaxation times.

The presence of such broad MM signals in proton spectra of the brain already was described in the early 1990s 6. Behar et al. assigned these MM resonances to cytosolic proteins, mostly to the methyl and methylene groups of protein amino acids. The signal of MMs in the range from 0 to 4.7 parts per million (ppm) consists of 10 individual peaks: 0.90 ppm (MM1); 1.21 ppm (MM2); 1.43 ppm (MM3); 1.67 ppm (MM4); 2.04 ppm (MM5); 2.26 ppm (MM6); 2.99 ppm (MM7); 3.21 ppm (MM8); 3.8 to 4.0 ppm (MM9); and 4.3 ppm (MM10) 7.

Because MM signals are very strong in short‐TE*/TE spectra, the omission of MM contributions in the fitting routine may yield substantial errors in the quantified metabolite levels 7. Moreover, the quantification of these strong MM signals, per se, may provide valuable information. MM concentrations were found to be age‐ and region‐dependent in the healthy brain 8, 9, 10. In addition, several studies have shown MM levels to be altered in various diseases 11, 12, 13. This makes them potentially valuable for clinical studies.

Many possibilities to properly handle MMs during quantification were proposed 7. In most cases, MMs only are accounted for to improve metabolite quantification. At lower field strengths ( ≤ 3T) and shorter TEs, in which MM resonances are mere bumps rather than distinct peaks, a mathematical estimation using a spline baseline is adequate 14, 15. At field strengths > 3T, such a simulation of the MM background usually is insufficient. Moreover, there has been an increased interest in MM quantification over the last couple of years. Previously published papers modeled the MMs at ultra‐high field with a single measured MM spectrum (typically a metabolite‐nulled MM spectrum acquired with inversion recovery methods) 10, 15, 16, 17, 18. These measured MM spectra are a well‐established MM model for metabolite quantification but do not allow for quantification of the individual MM peaks. Consequently, the quantification of metabolites with such a model only will fail when individual MM resonances are pathologically altered 13. Multiple molecules and chemical groups typically contribute to even single MM peaks, which impedes any effort for quantum‐mechanical simulations as applied for metabolite signals. Hence, we aimed to derive the individual MM basis spectra by parameterization of in vivo metabolite‐nulled spectra 13. If the MM basis spectra are combined with metabolite basis spectra in one basis set, MMs and metabolites simultaneously can be quantified from FID‐MRSI spectra.

The main goal of this work was to simultaneously map individual macromolecule components and metabolite levels in a healthy human brain at 7T. The information about the individual MM resonances may provide a better understanding of MM pathological changes in a diseased brain.

## METHODS

### Volunteers

Fourteen healthy volunteers (five females; 31 ± 4 years of age) were measured on a 7T whole‐body MR scanner (Magnetom, Siemens Healthcare, Erlangen, Germany) with a 32‐channel receive coil array combined with a volume transmit coil (NovaMedical, Wilmington, Massachusetts, USA). The study was approved by the institutional review board. Written informed consent was obtained from all subjects prior to the MR examination.

### Data Acquisition

A 3D, T_1_‐weighted, magnetization‐prepared, two rapid acquisition gradient echoes sequence 19 was acquired to position the MRSI slice in the region of interest and to derive the gray matter (GM)/white matter (WM) tissue maps. Standard field‐map‐based first‐ and second‐order B_0_‐shimming was performed on a shim volume with a slice thickness of 20 to 25 mm and full in‐plane brain coverage. Subsequently, a $B_{1}^{+}$‐map 20, 21 and B_0_‐map were acquired, which served for pulse‐power calibration and signal‐amplitude normalization.

Metabolite‐nulled spectra utilized for MM parameterization were acquired using double inversion recovery 2D‐FID‐MRSI with two inversion 40 ms Wurst pulses from six healthy volunteers (28 ± 2 years) with the following parameters 18, 22: repetition time ((TR), 879 ms; TE*, 1.3 ms; TI_1_, 570 ms; TI_2_, 21 ms; flip angle, 55°; field of view (FoV), 180 × 180 mm^2^; matrix size, 32 × 32; nominal voxel size, 5.6 × 5.6 × 12 mm^3^; 2,048 complex spectral data points; and acquisition bandwidth, 6,000 Hz. Metabolite residuals of N‐acetyl‐aspartate (NAA) (2.01 ppm), myo‐inositol (3.52 ppm), glutamate (Glu) (2.3 ppm), glutamine (Gln) (2.45 ppm), and total creatine (tCr) (i.e., Cr + phosphocreatine (PCr)) (3.98 ppm) were carefully removed from the metabolite‐nulled spectra using the AMARES algorithm 23 to minimize any metabolite contributions that could have influenced the parameterization.

The spectroscopic data were acquired with a single‐slice 2D‐FID‐MRSI sequence 3, with the following parameters: TR, 600 ms; TE*, 1.3 ms; flip angle, 45°; FoV, 220 × 220 mm^2^; nominal voxel size, 3.4 × 3.4 × 8 mm^3^; matrix size, 64 × 64; 2,048 complex spectral data points; acquisition bandwidth, 6,000 Hz; WET water suppression consisting of four 40 ms Gaussian pulses with a 110 Hz bandwidth optimized for 7T 4; one average with an elliptically sampled k‐space acquired in a pseudo‐spiral pattern; and scan time ∼30 min.

### Parameterization of the Metabolite‐Nulled Spectrum

Individual MM components in the range of ∼0.5 to 4.0 ppm (altogether nine separate peaks: 0.90 ppm (MM1), 1.21 ppm (MM2), 1.43 ppm (MM3), 1.67 ppm (MM4), 2.04 ppm (MM5), 2.26 ppm (MM6), 2.99 ppm (MM7), 3.21 ppm (MM8), and 3.77 ppm (MM9)) were considered for parameterization from the average metabolite‐nulled spectrum. The MM peak at 4.3 ppm (MM10) was not parameterized due to proximity to the water peak.

Two methods of parameterization implemented in jMRUI 5.2 were applied: 1) Hankel‐Lanczos singular value decomposition (HLSVD) 24; and 2) advanced method for accurate, robust, and efficient spectral fitting (AMARES) 23.

For modeling the individual MM peaks, 13 components were used in the HLSVD parameterization and nine Gaussian functions in the AMARES parameterization. A higher number of components in case of HLSVD was necessary to account for the baseline imperfections. These extra components, which did not represent the MM contribution, were removed afterward. In AMARES, the prior knowledge of the chemical shifts (adapted from 7, 25) was utilized in the initial phase of modeling. The amplitude, line width, and relative phase were adjusted in an iterative manner to achieve minimal fitting residuals.

### Basis Spectra of Metabolites

LCModel 6.3 26 was used to quantify all data acquired with a single‐slice 2D‐FID‐MRSI sequence. The metabolite basis set consisted of 17 simulated metabolite resonances: glucose; aspartate (Asp); total choline (tCho) (glycerophosphorylcholine + phosphorylcholine); tCr (PCr + Cr); γ‐aminobutyric acid (GABA); Glu; Gln; glutathione (GSH); glycine; lactate; myo‐inositol (Ins); NAA; N‐acetyl‐aspartyl glutamate (NAAG); scyllo‐inositol; and taurine (Tau). Simulations were performed in NMR Scope (jMRUI 5.0) using one hard pulse and a consecutive FID acquisition. The first 39 points of all simulated FIDs were removed to account for the first‐order phase error present due to the TE* of 1.3 ms. Consequently, seven different MM models (see below) were combined with the same metabolite basis spectra of 17 simulated chemical compounds to form seven complete basis sets. Finally, all the resultant seven different basis sets were used for data quantification.

### Making the Basis Sets With Different MM Models

The parameterized individual MM peaks were saved separately and included into seven different basis sets. With an increasing number of independent components in the basis set, the risk of over‐parameterization of the mathematical model used to quantify MRS data increases. Therefore, the number of degrees of freedom of our model was decreased either by grouping of components or by applying soft constraints on MM signal intensities.

The following MM models were created:

1 1. Full measured MM spectrum (full_MM)
2 2a. Individual MM components parameterized via HLSVD (ind_MM_HL)
3 2b. Individual MM components parameterized via AMARES (ind_MM_AM)
4 3a. Groups of individual MM components from HLSVD (grp_MM_HL)
5 3b. Groups of individual MM components from AMARES (grp_MM_AM)
6 4a. Individual MM components from HLSVD using soft constraints (con_MM_HL)
7 4b. Individual MM components from AMARES using soft constraints (con_MM_AM)

For MM models 4a and 4b, soft constraints were applied via concentration ratio priors (CRPs) using the LCModel parameter CHRATO. This approach is based on specifying prior probabilities on chosen signal intensity ratios of individual MM peaks. The MM1 (0.9 ppm) peak was used as a ratio denominator because it is easy to quantify and does not overlap with any metabolic resonance. First, every single MM peak was quantified from six measured metabolite‐nulled spectra using AMARES. From the quantification results, prior knowledge about a signal intensity ratio of MM(XX)/MM1 was determined (MM(XX) being the signal of MM peaks other than MM1), which then was included in the LCModel control file. The expected values of the ratios, together with their standard deviations, were optimized iteratively.

For MM models 3a and 3b, MM groups were defined as: MM1–MM4 (0.9–1.6 ppm); MM5–MM6 (2.04–2.26 ppm); MM7–MM8 (2.99–3.21 ppm); and MM9 (3.77 ppm), as previously proposed in 25. This grouping has no known biological or chemical relevance and solely was chosen to enable comparison to the previously published results 25.

Data acquired from 14 volunteers were quantified with these basis sets using LCModel 6.3 26 in the spectral range from 4.2 ppm to 0.2 ppm. In addition, lipid signals were simulated in basis set with *full_MM* using LCModel (parameter CHSIMU) to improve the handling of lipid resonances in the 1.2 to 1.4 ppm region originating from the extracerebral adipose and muscle tissue (see Supporting Fig. S1).

### Postprocessing

All measured data were processed with an in‐house developed program based on Bash 4.2.25 (Free Software Foundation, Boston, Massachusetts, USA) and MatLab R2009a (MathWorks, Natick, Massachusetts, USA) scripts 27, which allowed automated postprocessing with minimal user interaction. Coil combination and phase correction of individual channels was performed using MUSICAL 28. Tissue segmentation into GM, WM, and cerebrospinal fluid (CSF) was carried out using FAST (functional MRI of the brain (MRIB) automated segmentation tool) 29. To obtain the corresponding tissue volume contributions for each MRSI voxel, the high‐spatial‐resolution segmented images were Fourier‐transformed to k‐space and matched to the MRSI resolution/point spread function before converting the data back to image‐space 30.

Thorough quality assurance was carried out to rule out any lipid‐contaminated voxels or poor‐quality spectra. The maps of SNR, Cramer‐Rao lower bounds (CRLB), and line width (i.e., full width at half maximum (FWHM)) of NAA served this purpose. Spectra with CRLB_NAA_ > 30% and FWHM_NAA_ > 20 Hz were automatically excluded from the analysis. A further visual inspection was performed on spectra with only one of these quality criteria fulfilled. Typically, 1% to 3% of all spectra from one dataset were discarded.

### Comparison of MM Models for Spectral Fitting

To test the six basis sets with parameterized MMs, metabolite signal amplitudes were compared to the results obtained using the basis set with a measured MM spectrum (*full_MM*). First, the quantified metabolite intensities were averaged for every metabolite separately per volunteer and method. Afterward, repeated measures analysis of variance to account for the repeated testing of quantified intensities of multiple metabolites, as well as post‐hoc, Bonferroni‐corrected paired post‐hoc tests, were used to compare the metabolite signal intensities for every method.

A *P* value ≤ 0.05 was considered to indicate significant results. Due to the relatively small sample size and the large number of metabolites, no multiplicity correction was performed for multiple dependent variables.

Metabolite maps were created for individual MM peaks and metabolites covering the whole slice. Voxels with a CRLBs higher than 30% were not displayed on the maps.

The signal amplitudes of all MM peaks were correlated with the GM fraction to investigate GM/WM differences. For this analysis, voxels with a CSF tissue fraction higher than 20% and a CRLB higher than 30% were excluded.

## RESULTS

### MM Parameterization

**Figure 1:**
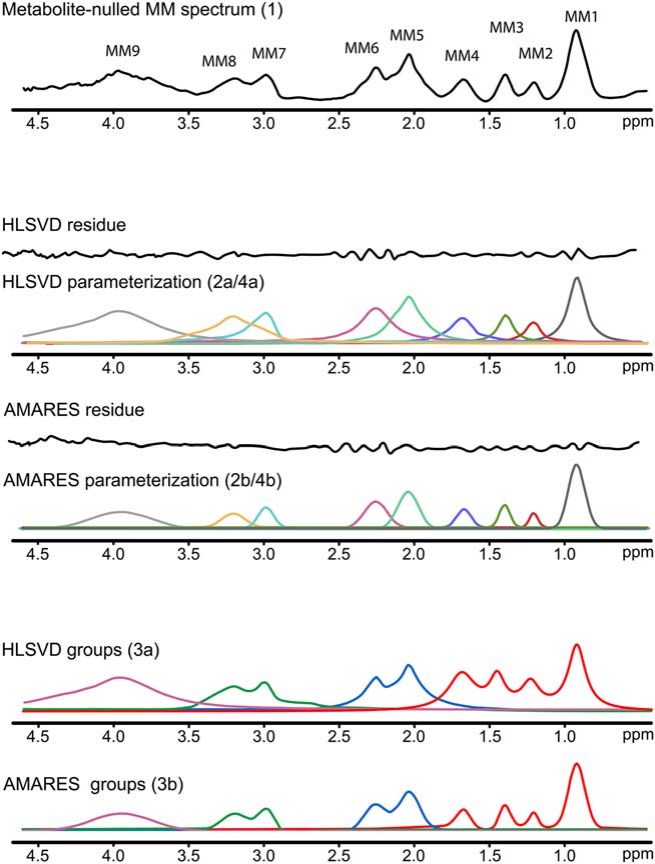
Parameterization of the metabolite‐nulled MM spectrum (top; MM model 1) utilizing two algorithms: HLSVD and AMARES (center: MM models 2a/4a and 2b/4b, respectively). Individual parameterized MM peaks were also combined into four MM groups (bottom: MM models 3a and 3b). AMARES, advanced method for accurate, robust, and efficient spectral fitting; HLSVD, Hankel‐Lanczos singular value decomposition; MM, macromolecule.

**Figure 2:**
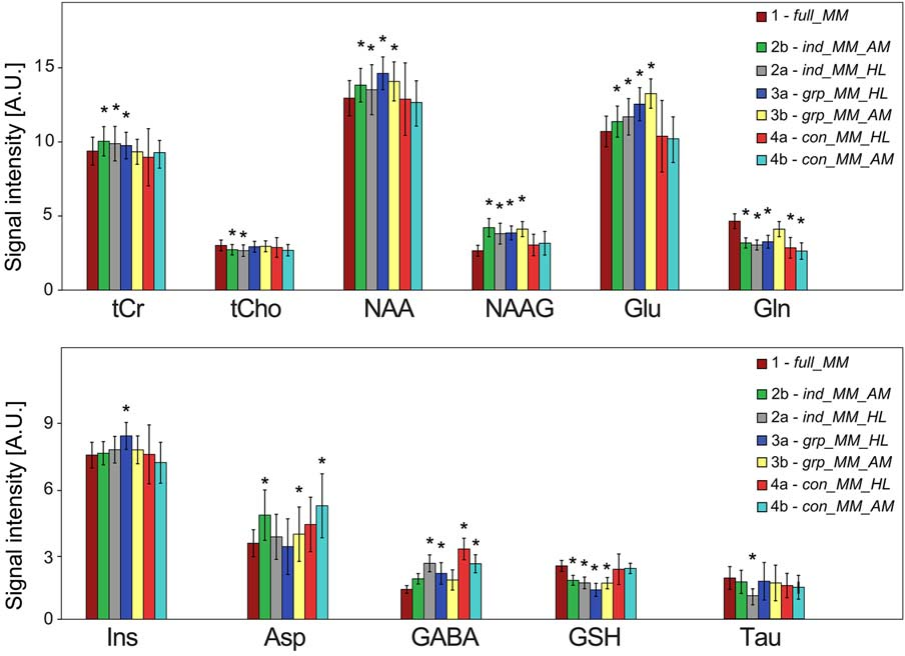
Comparison of metabolite signal amplitudes quantified using different basis sets. The results obtained using *full_MM* (MM model 1) were considered a gold standard and compared (repeated measures analysis of variance) to the remaining six basis sets (MM models 2a/b, 3a/b, and 4a/b). (* indicates P ≤ 0.05). The bar plots show the quantified mean metabolite signal intensities ( ± standard deviation) from all volunteers averaged over the whole MRSI slice. A.U., arbitrary unit; Gln glutamine; Glu, glutamate; MM, macromolecule; NAA, N‐acetyl‐aspartate; NAAG, N‐acetyl‐aspartyl glutamate; tCho, total choline; tCr; total creatine.

**Figure 3:**
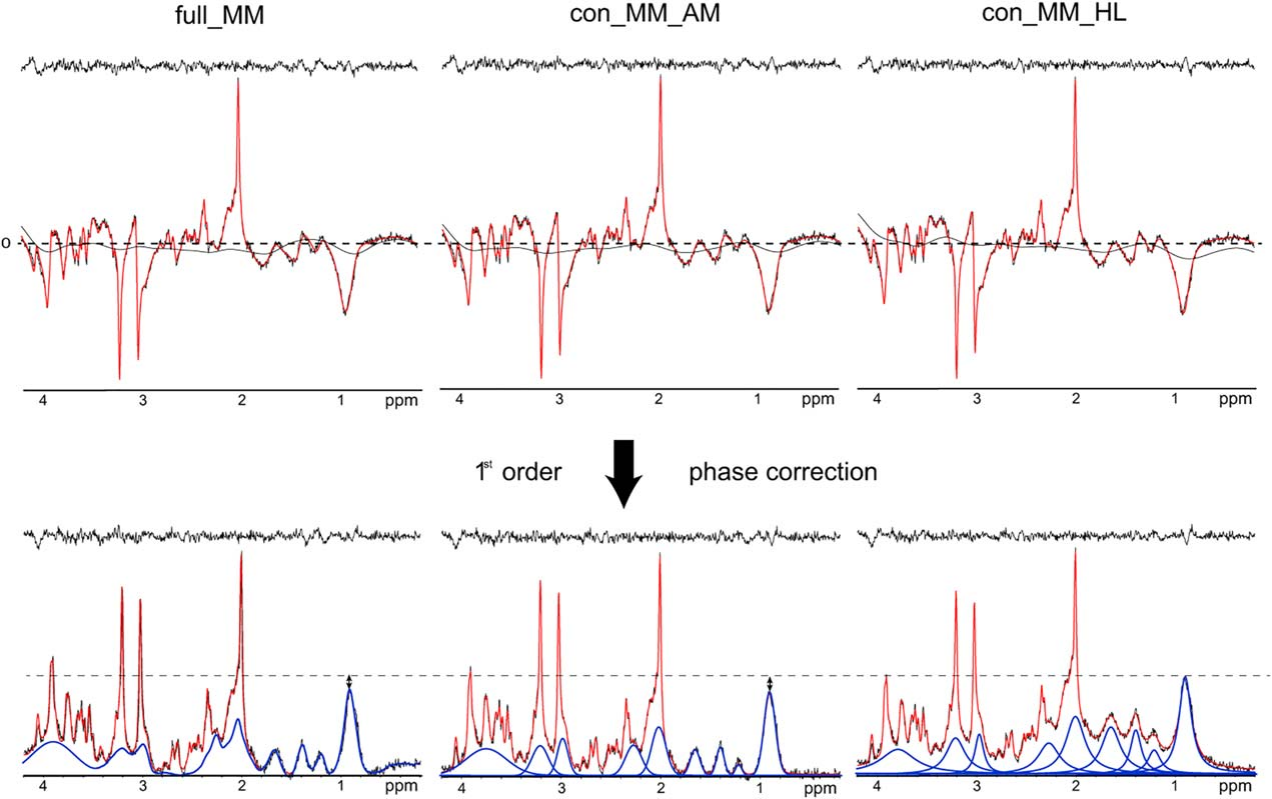
Top: An example of LCModel quantification with *full_MM, con_MM_AM*, and *con_MM_HL*, showing 2D‐FID‐MRSI spectrum (black) and fit (red) before first‐order phase correction. Bottom: A corresponding 2D free induction decay MR spectroscopic imaging spectrum (black), fit (red), and fitted MM (blue) quantified using *full_MM* 1, *con_MM_HL* (4a), and *con_MM_AM* (4b) after first‐order phase correction. An elevated baseline is visible in the case of con_MM_HL. The shown spectra originate from a single voxel located in the central part of the slice superior to the lateral ventricles (GM_fraction_ = 60%, WM_fraction_ = 39%) GM, gray matter; MM, macromolecule; ppm, parts per million; WM, white matter.

**Figure 4:**
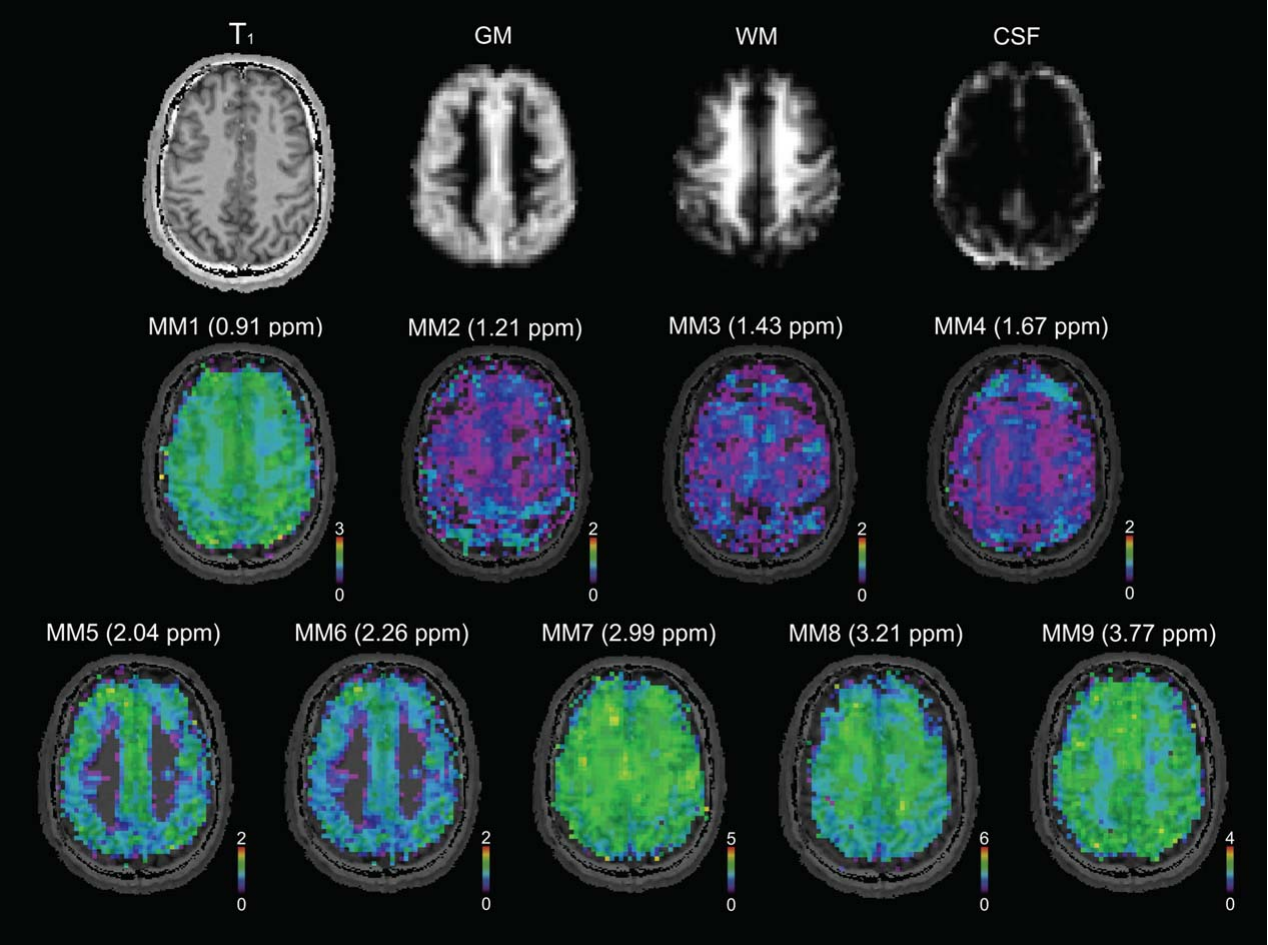
Maps of nine individual MM components (MM1–MM9) in native resolution (64 × 64 voxels) measured in a volunteer, are shown, together with tissue maps and a T_1_‐weighted image. Most of the MM resonances tend to be higher in GM compared to WM; only MM8 at 3.21 ppm is higher in WM CSF, cerebrospinal fluid; GM, gray matter; MM, macromolecule; ppm, parts per million; WM, white matter.

Both methods of parameterization (i.e., using AMARES and HLSVD) provided a good approximation of the measured MM spectrum in terms of a flat‐fitting residual (Fig. 1). A minimal number of components in HLSVD parameterization was found to be 13. An overall broader line shape of HLSVD parameterization (Figs. 1 and 2a, 4a) was reflected in the elevated baseline after the combination of individual MM resonances into MM groups (Figs. 1 and 3a).

### Comparison With an Established Method

The metabolite signal amplitudes obtained via the six parameterized basis sets were compared with the metabolite signal amplitudes obtained via the basis set that included the full measured MM spectrum, which can be considered a standard quantification approach (Fig. 2). Significant differences for most of the quantified metabolites were found if the number of degrees of freedom was not decreased (basis sets *ind_MM_HL* and *ind_MM_AM*) (Supporting Table S1). Data quantified with *grp_MM_HL* were significantly increased for NAA (16.03%), NAAG (18.60%), Glu (22.57%), Ins (19.39%), GABA (17.27%) (all *P* < 0.001), and tCr (5.84%) (*P* = 0.023), and were decreased for GSH (−28.92%) (*P* < 0.001) and Gln (−13.78%) (*P* = 0.007). Data quantified with *grp_MM_AM* were significantly increased for NAA (13.77%), NAAG (18.60%), and Glu (29.31%) (all *P* < 0.001), and were decreased for GSH (−18.57%) (*P* < 0.001), and Asp (−6.92%) (*P* = 0.018).

There were no significant differences for all the major metabolites if CRPs were imposed on both parameterized models (*con_MM_HL* and *con_MM_AM*) (Supporting Table S1). For *con_MM_HL*, Gln showed significantly decreased signal amplitudes (−25.41%) (*P* = 0.021) and GABA showed a significant increase (51.15%) (*P* = 0.004). For *con_MM_AM, Gln* was significantly decreased (−30.41%) (*P* < 0.001), whereas Asp (36.63%) (*P* < 0.001) and GABA (39.88%) (*P* = 0.014) showed a significant increase.

Figure 3 illustrates the quantification with *full_MM, con_MM_AM*, and *con_MM_HL*. The flat fit residuals and spline baselines were comparable for all three approaches; however, the elevated baseline of *con_MM_HL* compared to *full_MM* and *con_MM_AM* after first‐order correction reveals the bias in MM quantification using this model. Therefore, the basis set with AMARES‐modeled MMs was considered a more suitable model for the MM background.

### Metabolic and MM Maps

**Figure 5:**
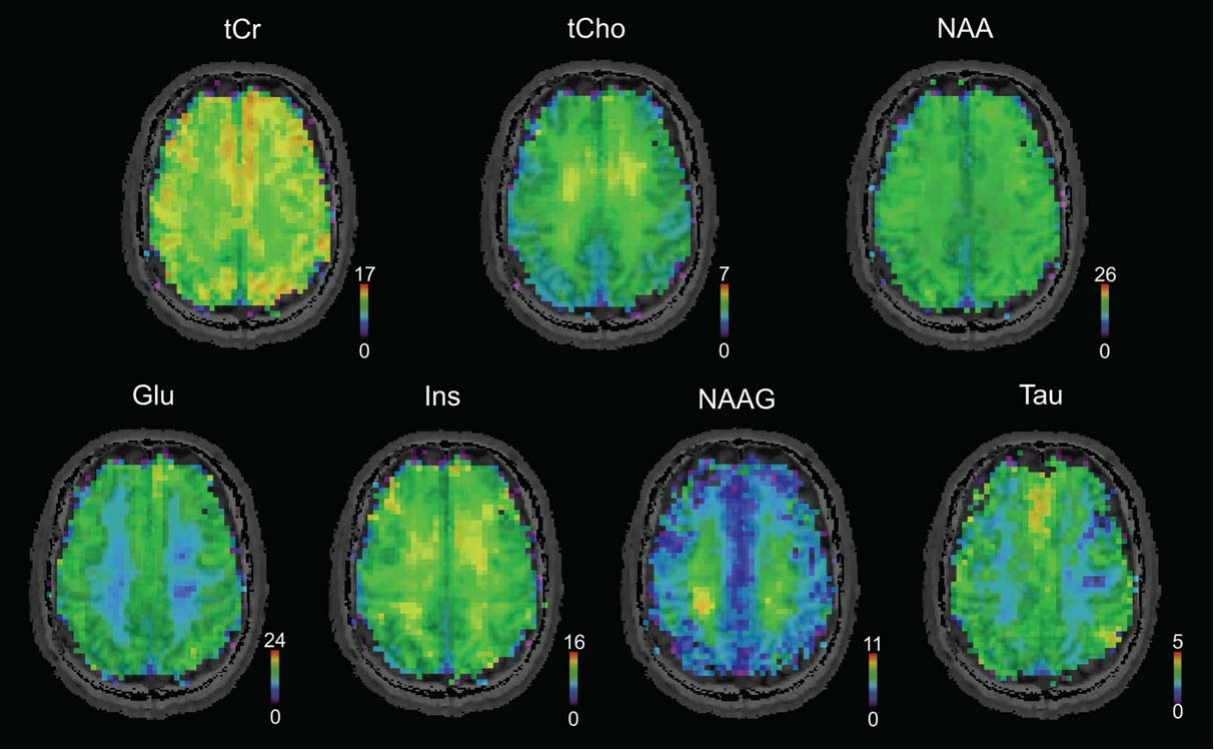
Metabolic maps in native resolution (64 × 64), measured in the same volunteer as in Figure 4. Seven metabolites were reliably quantified with Cramer‐Rao lower bounds below 30% in all volunteers. Physiological gray matter/white matter and spatial differences are visible. Glu, glutamate; Ins, myo‐inositol; NAA, N‐acetyl‐aspartate; NAAG, N‐acetyl‐aspartyl glutamate; tCho, total choline; tCr; total creatine.

**Figure 6:**
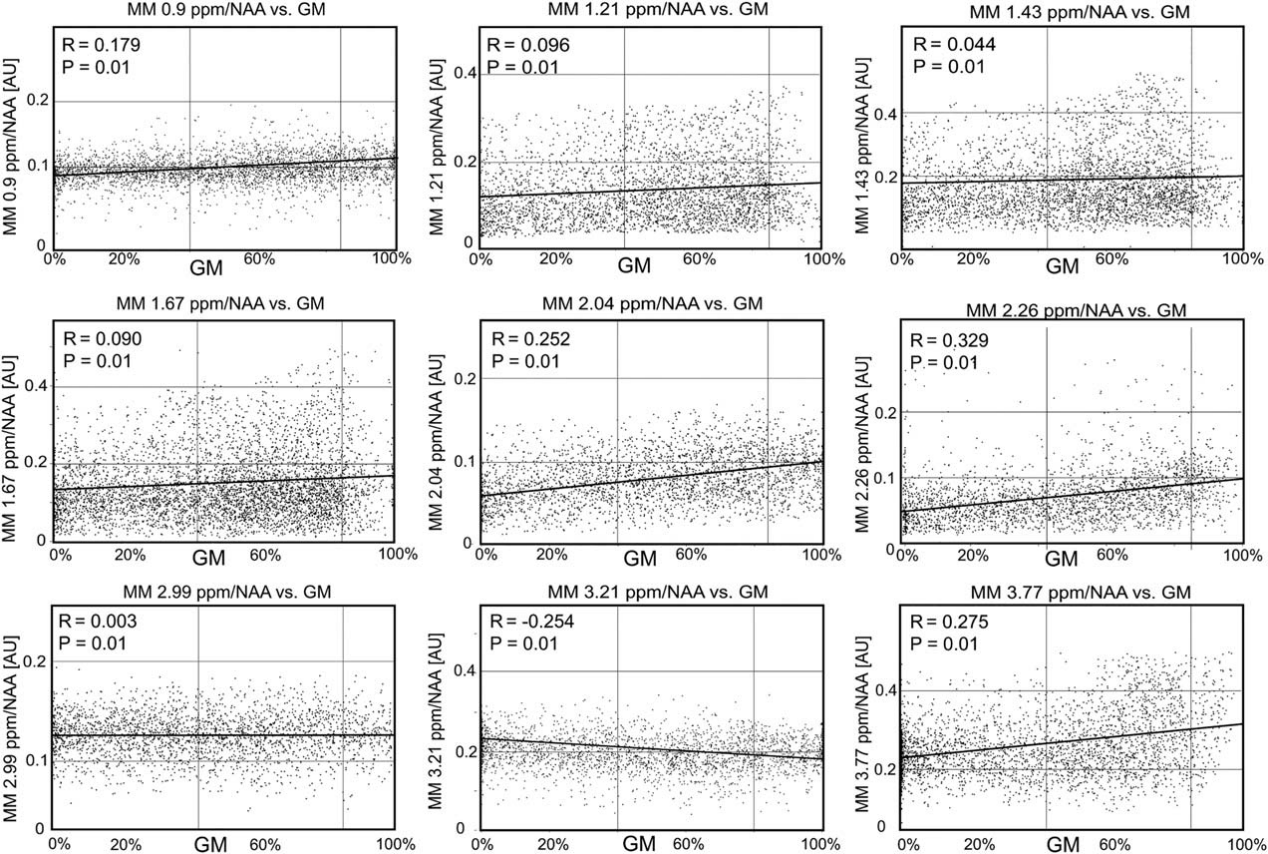
Scatter plots summarizing the dependence of the macromolecule peaks on the gray matter fraction. Data from all volunteers were used. The lines of best fit for a linear regression model are shown in black. AU, arbitrary unit; GM, gray matter; MM, macromolecule.

The simultaneous quantification of the acquired 2D‐FID‐MRSI with AMARES‐modeled individual MMs with CRPs enabled the mapping of metabolites and MMs within one measurement (Figs. 4, 5). Physiological tissue differences were found in both MM (Fig. 4) and metabolite levels (Fig. 5). Figure 4 and Figure 6 illustrate that most individual MM resonances tend to be higher in GM compared to WM. MM7 at 2.99 ppm appeared to be relatively evenly distributed over the whole brain slice. In contrast, MM8 at 3.21 ppm was higher in WM compared to GM. The signals of Glu and tCr were higher in cortical GM compared to WM, whereas the signal of NAAG was two times higher in mesial WM compared to cortical GM. tCho exhibited the highest signal intensity in the frontal WM area. Minimal tissue or regional differences were observed for NAA. A low‐abundant metabolite tau was reliably mapped with CRLB < 30% and found to be higher in GM. No visual difference was found between the metabolic maps obtained from quantification with *full_MM* and *con_MM_AM*.

## DISCUSSION

In this study, we improved quantification of in vivo 2D‐FID‐MRSI spectra by including information about individual macromolecular peaks in the fitting prior knowledge. Thus, we were able to simultaneously map the spatial distribution of individual MM components together with metabolites that resonated between 4.2 and 0 ppm.

### Comparison to Previous Studies

The number of MM model components that are necessary for parameterization is influenced by the actual chemical shift dispersion and the SNR of the parameterized spectrum. In a pioneering work by Seeger et al. 13, only four MM components were sufficient to adequately model the MM contribution in human brain spectra obtained at 1.5T.

Unlike the metabolite spectra, a plethora of chemical compounds contribute to every single MM peak. These individual contributions cannot be resolved at 7T but are some of the strongest factors that influence the MM lineshape. In addition, a local susceptibility broadening affects the lineshape as well. Recently published single‐voxel animal studies 25, 31 parameterized the MM background using LCModel built‐in Gaussian functions with precisely defined chemical shifts, linewidths, and amplitudes. Lopez‐Kolkovsky et al. 25 parameterized MM from a metabolite‐nulled rat spectrum acquired at 17.2T, with a total of 32 individual MM resonances. However, Lee and Kim 31 aimed to parameterize the MM contribution directly from a rat short‐TE spectrum at 9.4T with a total of 25 MM components. Snoussi et al. 32 fitted MM from the human brain using the sum of 19 Gaussian functions. The presence of the additional MM resonances had to be handled to avoid the model overparameterization, either by combining individual peaks into groups 25 or by merging the peaks into a single model MM spectrum 31, 32.

Nine to 10 distinct MM peaks can be distinguished in the human brain at magnetic fields close to 7T 7, 18, 33, although recent reports have hypothesized another MM resonance around 2.6 ppm 34, 35. We parameterized an averaged metabolite‐nulled spectrum by AMARES using nine Gaussian functions and by HLSVD using 13 components. HLSVD allows only minimal control over the fitting output. Moreover, the input number of components affects the fitting result. Therefore, to properly handle the baseline of the parameterized metabolite‐nulled spectrum, a minimum of 13 components were required to achieve flat residuals. Afterward, the four redundant resonances that did not correspond to MM peaks were removed from the results. AMARES, however, is a more interactive tool for which prior knowledge of the quantified peaks is required. Hence, the parameterization may be well‐controlled and iteratively improved. The prior knowledge for AMARES was adapted from that proposed in previous studies 25, 31.

### Comparison With an Established Method

A single measured MM spectrum was considered an established method of handling MM in vivo and served as a gold standard for all our comparisons. Nevertheless, any systematic deviations of our gold standard from the real values could not be estimated and were not the subject of this work.

To test the simultaneous quantification of metabolites and macromolecules, the metabolite signal amplitudes of the gold‐standard method were compared to the six methods using parameterized MMs. It is obvious from Figure 2 that the inclusion of additional components into the basis set without using any constraints (*ind_MM_AM* and *ind_MM_HL*) led to significant overestimation or underestimation of the metabolites. Thus, two different methods were examined by decreasing the degrees of freedom of the MM. Grouping of the MM peaks, as described in 25, lead to significant overestimation or underestimation of several metabolite signal intensities compared to the gold standard, even in higher‐abundant metabolites, such as NAA, Glu, and Ins, for both parameterization methods. A possible explanation may lie in the accuracy of the modeled MM peaks. As stated above, any MM model is a simplification of the real world in vivo MM resonances. Our MM models involve a certain unknown deviation from in vivo MM. Several modeled MM peaks were added together to form MM groups. Thus, the error in the model might have increased, rendering significant differences. In our case, the application of CRPs on the MM peaks appeared to be a more robust way to handle the over‐parameterization. Furthermore, the information about all MM peaks was preserved. Despite elevation of the baseline in the case of *con_MM_HL* (Fig. 3), most of the metabolite levels remained unchanged for both parameterization approaches. Only some low‐abundant metabolites showed a significant difference compared to results obtained via the *full_MM* model. Nevertheless, the HLSVD parameterization did not yield a valid MM model, which was manifested by the elevated baseline and broadened lineshapes of the MM peaks (Fig. 3). The HLSVD is an algorithm based on singular value decomposition with minimal prior knowledge. It was shown to be a powerful water removal tool 36. However, for more complex tasks with several overlapping resonances, it often fails to provide reliable results 37. Craveiro et al. 37 reported significant differences between the metabolite residuals removal of the metabolite‐nulled MM spectrum using HSLVD and AMARES. In our study, HLSVD did not successfully model the MM peaks using only nine components. To minimize the fitting residual, 13 HLSVD components had to be input, and the four additional components were later discarded. AMARES algorithm outperformed HLSVD and therefore is recommended for the parameterization. All the presented maps were created only from the results obtained with *con_MM_AM*.

### Metabolic and MM Maps

Spatial maps of nine MMs, together with seven metabolites, were mapped in all volunteers in approximately half an hour. To our knowledge, this is the first time that the individual macromolecular contributions were mapped with such a high spatial resolution (64 × 64 matrix size).

The physiological background of the observed GM/WM and regional MM differences is unclear, which also is connected to the contribution of various chemical compounds to a single MM peak. The MM1 (0.9 ppm) contains signals of the protein amino acids leucine, isoleucine, and valine; MM2 (1.21 ppm) and MM3 (1.43 ppm) threonine and alanine; MM4 (1.67 ppm); and MM7 (2.99 ppm) lysine and arginine 6. The contribution of Gln and Glu in MM5 (2.04 ppm) and MM6 (2.26 ppm) 6 may be one of the causes of higher GM signal for these two MM resonances. The previously reported higher signal of MM (overall) in GM compared to WM 10, 18 was confirmed for all MM resonances except for MM7 (2.99 ppm), which was evenly distributed; and MM8 (3.21 ppm), which was higher in WM. One possible explanation is a different origin of MM8. In contrast to the other MM peaks, MM8 was not found in in vitro dialyzed cytosol 6. Based on this, we hypothesize that MM8 probably originates from the cell membrane or the extracellular area.

The MM maps enable the visualization of even subtle spatial and tissue differences, which may be clinically interesting for various brain diseases. Hwang et al. 38 and Graham et al. 12 showed maps of the MM compound resonating at 1.3 ppm in stroke patients, although a mixed signal of MMs and lipids was detected. Changes in MM also were observed in patients with multiple sclerosis. The MM at 0.9 ppm and 1.3 ppm were elevated in acute lesions compared to those in chronic lesions or in controls 11. A more recent study 39 reports significantly decreased signals of MM (including lipids) at 0.9, 1.2 to 1.4, and 2.0 ppm in chronic lesions and normal‐appearing WM compared to cortical gray matter. The above‐mentioned MM differences often are connected to a particular MM peak and do not manifest over the whole spectral range. A quantification of patient data with only a single measured MM background in the basis set leads not only to incorrect estimation of MMs but also to biased metabolic quantification. In addition, a rather diffuse MM change, such as that observed in 39, favors utilization of MRSI over single‐voxel spectroscopy.

The spatial and tissue differences of metabolites found in our study were in good agreement with previous studies 1, 4, 40, 41. An increased signal of tCho in the mesial frontal area has been reported previously 4, 40, as well as the strong GM/WM contrast in tCr and Glu maps 4, 18, 42. NAA and NAAG both are neuronal compounds 43, and their concentration should be similar in both cell bodies (GM) and axons (WM). The discrepancy observed for NAAG in multiple MRS studies 18, 42, 44, as well as in this work, thus far has not been satisfactorily explained.

### Limitations

A truly quantitative comparison of the different MM models was not possible because the established method fits MMs with a single MM spectrum, whereas the parameterized MM models use different numbers of separate resonances, which cannot be statistically compared. Therefore, only the effect of the MM models on the quantification of metabolites was quantitatively estimated.

In a diseased brain, the changes in MM levels frequently are accompanied by changes in lipids. Moreover, lipid contamination due to the point spread function and subject movement, particularly in the region from 1.2 to 1.4 ppm, is very challenging and requires proper lipid handling even in a healthy brain, as can be seen in MM2, MM3, and MM4 maps (Fig. 4). The occipital part in the MM2 and the frontal part in the MM4 maps may be contaminated with the extracranial lipid signal. An additional lipid model included in the basis set may reduce this problem, but also may cause a further unwanted overparameterization. The proposed model of the MMs may be improved by parameterization using a higher number of components per MM peak to achieve a nonideal (i.e., non‐Gaussian, non‐Lorentzian, non‐Voigt) lineshape, and the lipid suppression could be further improved 45.

The use of a single‐component MM spectrum is a much more conservative MM model and provides a fast and robust way to handle MM in short‐TE/short‐TE* spectra. The process of determining the optimal constraints for a parameterized MM model is a lengthy and delicate procedure, especially when a MM peak overlaps more than one metabolic resonance. Yet, if the quantified spectra are more demanding (i.e., lipid contamination, pathological changes), the parameterized individual MM peaks with soft constraints provide better results. Furthermore, mapped MM resonances may serve as a biomarker for pathological changes in the brain metabolism.

## CONCLUSION

We have mapped a total of nine macromolecular resonances and seven metabolites in the healthy human brain by simultaneous quantification from 2D‐FID‐MRSI spectra. AMARES provides an efficient way to parameterize macromolecules from the metabolite‐nulled in vivo spectrum. The improved model of macromolecules derived by parameterization of the metabolite‐nulled spectrum may facilitate the detection and spatial mapping of pathologically altered macromolecules.

## Supporting information

Additional supporting information may be found in the online version of this article.

**Fig. S1.** The representative ^1^H‐FID‐MRSI spectra chosen from four different brain regions (A‐D) shown for the quantification without lipid handling and with lipids included into the spectral fitting prior knowledge. The lipid resonances were simulated using LCModel (parameter CHSIMU). *full_MM* was used as MM model for both quantification, with and without lipid handling. For approximately 80% to 85% of the voxels, the contamination with extracerebral lipids was minimal (spectra A and B). Spectra in proximity to the skull region (e.g., spectrum C) had fewer metabolites fitted with CRLBs below the given threshold if no lipid information was included into the prior knowledge. In some cases (typically 1%–2% of all spectra), the lipid handling failed and lipids were not fitted even for the basis set with lipid handling (e.g., spectrum D). However, these voxels are often ruled out due to other reasons (such as increased line widths, huge CSF contribution, etc.).
**Table S1.** The most important statistical measures for comparison of metabolite signal amplitudes quantified using different basis sets (Fig. 2). The results obtained using full_MM (MM model 1) were compared using repeated measures ANOVA to the remaining six basis sets (MM models 2a/b, 3a/b, and 4a/b).
