# Supplementary material for: Simultaneous mapping of metabolites and individual macromolecular components via ultra‐short acquisition delay 1H MRSI in the brain at 7T
Source: Magn Reson Med. 2017 Jun 22;79(3):1231–40. doi: 10.1002/mrm.26778 (PMC5811892; doi:10.1002/mrm.26778)
Supplement: Supplementary file 1 — Fig. S1. The representative 1H‐FID‐MRSI spectra chosen from four different brain regions (A‐D) shown for the quantification without lipid handling and with lipids included into the spectral fitting prior knowledge. The lipid resonances were simulated using LCModel (parameter CHSIMU). full_MM was used as MM model for both quantification, with and without lipid handling. For approximately 80% to 85% of the voxels, the contamination with extracerebral lipids was minimal (spectra A and B). Spectra in proximity to the skull region (e.g., spectrum C) had fewer metabolites fitted with CRLBs below the given threshold if no lipid information was included into the prior knowledge. In some cases (typically 1%–2% of all spectra), the lipid handling failed and lipids were not fitted even for the basis set with lipid handling (e.g., spectrum D). However, these voxels are often ruled out due to other reasons (such as increased line widths, huge CSF contribution, etc.). Table S1. The most important statistical measures for comparison of metabolite signal amplitudes quantified using different basis sets (Fig. 2). The results obtained using full_MM (MM model 1) were compared using repeated measures ANOVA to the remaining six basis sets (MM models 2a/b, 3a/b, and 4a/b). [file MRM-79-1231-s001.docx]

**Supporting Material**


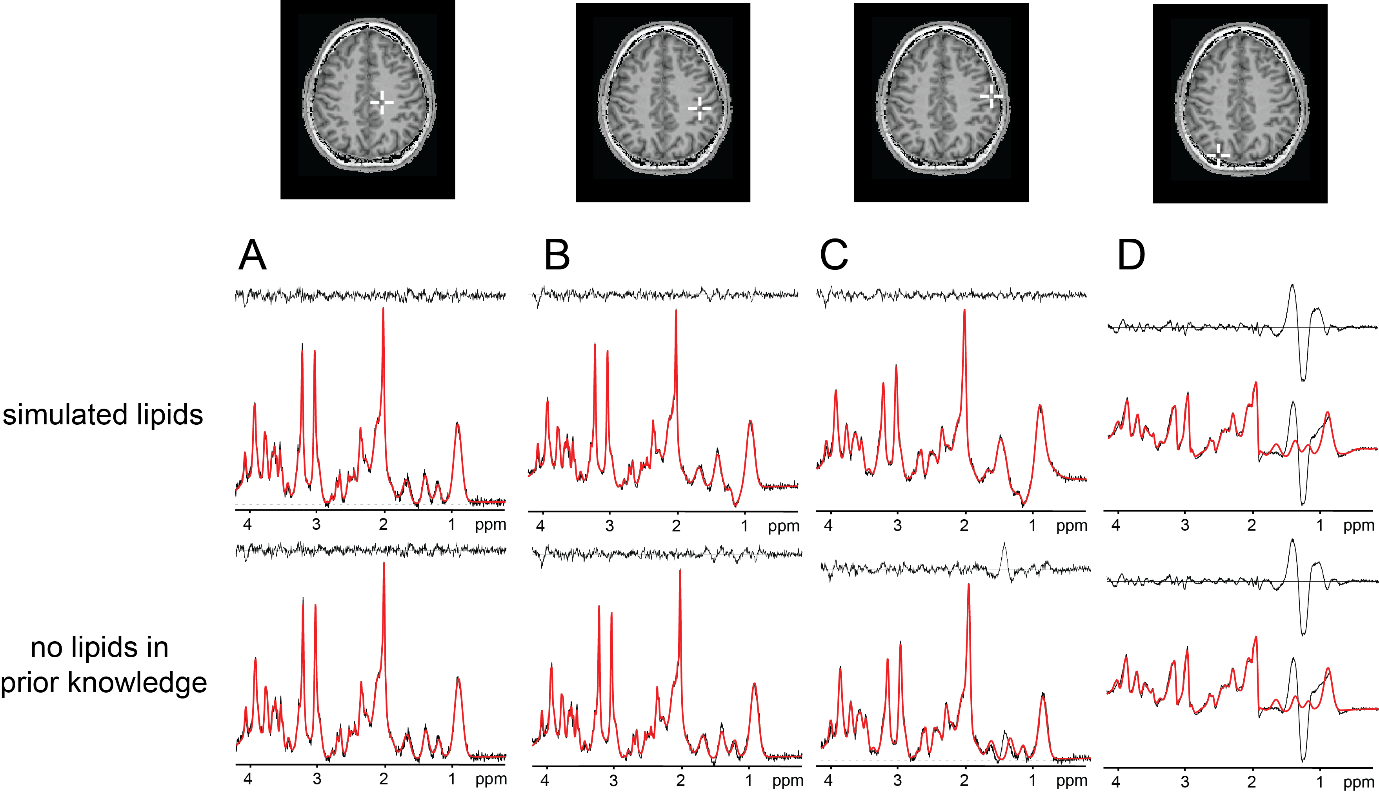


Supporting Figure S1 – The representative ^1^H-FID-MRSI spectra chosen from four different brain regions (A-D) shown for the quantification without lipid handling and with lipids included into the spectral fitting prior knowledge. The lipid resonances were simulated using LCModel parameter CHSIMU. *full_MM* was used as MM model for both quantification, with and without lipid handling. For approximately 80-85% of the voxels, the contamination with extracerebral lipids was minimal (spectra A and B). Spectra in proximity to the skull region (e.g. spectrum C) had fewer metabolites fitted with CRLBs below the given threshold if no lipid information was included into the prior knowledge. In some cases (typically 1-2% of all spectra), the lipid handling failed and lipids were not fitted even for the basis set with lipid handling (e.g. spectrum D). However, these voxels are often ruled out due to other reasons (such as increased line widths, huge CSF contribution, etc.).

Supporting Table S1 –The most important statistical measures for comparison of metabolite signal amplitudes quantified using different basis sets (Fig. 2). The results obtained using full_MM (MM model 1) were compared using repeated measures ANOVA to the remaining six basis sets (MM models 2a/b, 3a/b, and 4a/b).

Note that the mean differences were obtained via subtracting the reference result from the compared basis set results (i.e. negative mean difference refers to an increase of the metabolite signal intensity compared to the reference.

| *. The mean difference is significant at the .05 level. |
| --- |
| b. Adjustment for multiple comparisons: Bonferroni. |
